# Mediating Factors in Nursing Competency: A Structural Model Analysis for Nurses’ Communication, Self-Leadership, Self-Efficacy, and Nursing Performance

**DOI:** 10.3390/ijerph17186850

**Published:** 2020-09-19

**Authors:** Ae Young Kim, In Ok Sim

**Affiliations:** Red Cross College of Nursing, Chung-Ang University 84 Heukseok-ro, Dongjak-Gu, Seoul 06974, Korea; springbreez@naver.com

**Keywords:** structural model, clinical nurse, communication ability, self-leadership, self-efficacy, nursing performance

## Abstract

This study examined the structural relationship among clinical nurses’ communication ability, self-leadership, self-efficacy, and nursing performance. A structural model analysis was applied to identify factors influencing nursing performance and analyze the effects of self-leadership and self-efficacy as mediators. A survey was conducted among clinical nurses working in general hospitals in Seoul, Gyeonggi, and Gangwon Province of the Republic of Korea. In the final analysis, data from 168 questionnaires were used. SPSS 24.0 and Amos 23.0 programs were used for frequency analysis, exploratory factor analysis, reliability analysis, correlation analysis, confirmatory factor analysis, structural equation model analysis, and mediating effect analysis through bootstrapping. The significance level was set at 5% for all analyses. First, the model’s fitness figures met the criteria for the appropriate judgment presented in previous studies, so the model between nurses’ communication ability, self-leadership, self-efficacy, and nursing performance was suitable for predicting a causal relationship. Second, the relationship between nurses’ communication ability and self-leadership had a statistically significant effect. Also, the relationship between communication ability and self-efficacy had a statistically significant effect. Third, nurses’ communication ability affected nursing performance through self-efficacy.

## 1. Introduction

### 1.1. Significance of the Study

Economic growth and interest in global health have led to a demand for quality improvement in health care. In particular, medical institution evaluation and certification investigations began, in November 2010, to reconsider the quality of medical institutions [1]. The medical institution certification system is intended to improve patient safety and quality of care. It also aims to provide high-quality medical services to medical consumers by inducing voluntary and continuous improvement efforts [1].

Due to these changes in the external environment, medical institutions are making great efforts to meet the needs of high-quality medical services and medical consumers [2,3]. Doctors and nurses are at the heart of human services that have a direct impact on healthcare consumer satisfaction. As the needs of patients become diverse and complex due to these changes in the social environment, hospitals are striving to innovate management methods through recent changes in medical services. In particular, nurses are the most important people required to understand the patient’s problems and to address and meet their needs. Therefore, the hospital operation strategy for this is focused on improving the nursing level through the competency development of nurses [4]. This is because high-level nursing not only effectively fulfills responsibilities and roles for patients, but also satisfies the needs of patients [2,5], and consequently contributes to achieving the goals of hospital institutions. Therefore, developing and strengthening the competence of these nurses is very important, and therefore it is very important to pay attention to expand nursing expertise qualitatively and quantitatively [6,7].

Concepts included in the competency of a nurse generally include factors such as nursing performance, communication skills, self-leadership and self-efficacy, and problem-solving skills. Ko [8] presents the nursing performance ability as the most important concept included in nursing competency. The nursing performance ability were classified into four sub-areas: performance, attitude, problem-solving ability, and application of nursing process. In addition, they argued that if the performance in these areas is evaluated to improve nursing performance ability, nurses in charge of patients can actively cope with rapidly changing environments and develop competitive competencies [5]. Communication ability is also a very important factor in nurse competency. The hospitals where nurses work are large and subdivided, and when performing patient management, insight and intuition are required in the process of understanding and solving patient problems. In particular, it is necessary to have communication skills in order to coordinate doctors and patients, educate patients and caregivers, and maintain cooperative relationships with other medical personnel. Moreover, communication is an important factor in the quality of nursing services and determines the dynamics and effectiveness of an organization [2]. That said, proper communication between nurses can be the best way to compensate and smoothly resolve any problems that may arise among hospital staff. A nurse’s ability to communicate in the workplace is an important factor in determining an organization’s performance [9]. In addition, communication skills and self-leadership are known to have a positive effect on nursing performance [9,10]. Providing adequate services requires communication skills during nursing [2,4,11]. Communication ability refers to the ability to communicate effectively and includes both appropriate and effective means of communication. It also includes the motivation to act appropriately and effectively with those involved in the conversation [12]. A nurse’s communication skills include the ability to understand patients, help with problem solving, and use clinical judgment logically [13,14]. As described above, practical communication skills include the ability to develop nursing skills by deeply grasping the patient’s needs and understanding the patient [15,16,17].

Choi et al. [5] also explained that self-leadership can affect nursing skills. This can affect an individual’s behavior or thoughts and is an important factor for nurses, who have to meet the needs of many patients. In the case of self-leadership, unlike general leadership, it is a power that influences the individual’s ego to improve the individual’s ability and provide flexible nursing practice. In addition, many studies have suggested that self-leadership can influence self-efficacy [6,18]. Self-efficacy is a belief in the ability to successfully perform any action with confidence to achieve results [19]. Park et al. [19] explained that healthcare workers are anxious that patients may experience problems during the treatment process, which can affect self-efficacy. Therefore, research into the self-efficacy of nurses is an important factor in enhancing nursing ability. Communication skills and self-leadership are also important.

To date, studies on factors affecting nursing competency have been very fragmented. Although there have been studies on whether nurses’ communication ability and self-leadership directly affect nursing performance, there have been few empirical studies on the mediating effects of self-leadership and self-efficacy between communication ability and nursing performance. It is essential to study psychological factors such as self-efficacy that can help improve performance [20]. This study examined the structural relationship between nurses’ communication ability, self-leadership, self-efficacy, and nursing performance. We anticipate that this study will help to analyze the mediating role of psychological factors such as self-leadership and self-efficacy in the relationship between communication ability and nursing performance. The results will provide insights to help better understand and improve the situation of nurses, who are at the heart of hospital services, and make the hospitals manage them efficiently.

### 1.2. The Purpose of the Study

The purpose of this study was to establish a model showing the structural relationship between communication ability, self-leadership, self-efficacy, and nursing outcomes as factors of the nurse’s competency, which are necessary for high-level patient care, and to verify the data empirically. In addition, the mediating effects of self-leadership and self-efficacy on the relationship between communication ability and nursing performance would be verified. The specific research goals for this study are as follows:

1. Establish a causal model among the nurses’ communication ability, self-leadership, self-efficacy, and nursing performance;
2. Investigate the relationship between practitioner nurses’ communication ability, self-leadership, self-efficacy, and nursing performance;
3. Examine the mediating effects of self-leadership and self-efficacy on the relationship between communication ability and nursing performance.

## 2. Materials and Methods

### 2.1. Study Participants

This study examined a population of nurses working in general hospitals, and used a convenience sampling method among the non-probability sampling methods. A survey was conducted with nurses working at the 2nd and 3rd general hospitals in S, G and Gang provinces, South Korea. This study was conducted after receiving approval from the CAU institutional review board (1041078-201911-HR-351-01).

The pilot survey was conducted from 13 January to 18 January 2020, and was conducted with 50 nurses working in general hospitals. The questionnaire survey was reviewed in the pilot survey, and the final questionnaire was prepared by accurately correcting and supplementing the content for the items that respondents were confused by. The survey was conducted from 17 February to 18 April 2020, and a questionnaire was prepared and distributed to nurses working in the second and third hospitals located in S, G and Gang city of South Korea. The appropriate sample size was calculated using the G*Power 3.1.9.1 program. In this study, the minimum sample size was 166, which was determined for regression analysis by fixing effect size = 0.15, Aapha error = 0.05, Power(1-β err prob) = 0.90, number of predictors = 14. The minimum number of subjects was 166, but 250 copies of the questionnaire were distributed, considering the expected dropout rate. Of the distributed questionnaires, 214 were collected, and 168 were used for the final analysis. The researchers excluded questionnaires that were incomplete or outliers. The distribution of demographic characteristics of the participants is shown in Table 1.

### 2.2. Research Instruments

#### 2.2.1. Composition of Research Tools

The questionnaire was the primary research tool used in this study. Based on the results of previous studies, the questionnaire collected information on demographics, the general characteristics of the participants, and nurses’ communication ability, self-leadership, self-efficacy, and nursing performance. The number of questions consisted of a total of 68 items, including 9 items on demographic and general characteristics, 16 items on communication ability, 13 items on self-leadership, 16 items on self-efficacy, and 14 items on nursing performance. Except for demographic characteristics, each item was scored using a Likert 1–5 point scale.

#### 2.2.2. Validity and Reliability of Research Tools

Exploratory factor analysis was conducted to determine the validity of the research tools in this study. In addition, Cronbach’s alpha, which shows the internal consistency, and item analysis was used to investigate the reliability.

The principal component analysis was performed for factor analysis, and the method of factorization was used for factor extraction with the eigen values greater than 1 and the method of specifying the number of factors. Cronbach’s alpha was used as a criterion for evaluating reliability. Generally, the reliability criterion Cronbach’s alpha has a value between 0 and 1, and is usually a reliable level if the coefficient is 0.6 or higher.

#### 2.2.3. Communication Ability

Communication skills are the ability to interpret other people’s conversations, express yourself, communicate actively, and understand from other people’s perspectives. In this study, a total of 20 items were composed based on the research of Hur [12], Cho and Park [21] to measure communication ability. When analyzing the communication ability of nurses, items 3, 8, and 12 with low factor loading were removed and analyzed again. The factor analysis resulted in the extraction of four factors, namely, interpretation ability, self-expression ability, leading communication, and understanding others’ perspectives. Table 2 shows the results of the factor analysis of communication ability and reliability.

#### 2.2.4. Self-Leadership

Self-leadership must have its own action-oriented strategy and can be explained by self-reward strategy and constructive and positive thinking. In this study, 13 questions were included to measure self-leadership based on the research of Neck and Houghton [22], Kim and Jyung [23], and Lee and Hong [24]. When analyzing self-leadership factors in nurses, items 1 and 5 with low factor placement were removed and analyzed again. As a result of factor analysis, three factors were extracted and named as constructive thinking, self-rewarding strategies, and action-oriented strategies. Table 3 shows the results of the factor analysis of self-leadership and reliability.

#### 2.2.5. Self-Efficacy

Self-efficacy refers to the ability of nurses to perceive that they can do it on their own, and this can be explained by including the ability to acquire knowledge and clinical skills, the ability to communicate with patients, and the ability to maintain nursing ethics. The self-efficacy questionnaire was amended and supplemented based on research by Park et al., (2009), and comprised 16 questions [25]. When analyzing the factors of self-efficacy of nurses, items 1, 5, 7, 8, and 13 with low factor loading were removed and analyzed. As a result of factor analysis, three factors were extracted and grouped into the categories knowledge and clinical skills, communicating with patients, and nursing ethics. The results of the factor analysis of self-efficacy and reliability are shown in Table 4.

#### 2.2.6. Nursing Performance

Nursing performance ability refers to the integrated ability required in the process of nursing work., and refers to the ability to assess and solve patient problems as well as the application and performance attitude of the nursing process, which is important for nursing. In this study, based on the research by Van de Ven and Ferry [26] and Ko [8], the nursing performance questionnaire was prepared and comprised 14 questions. When analyzing the factors of nursing performance of nurses, the questionnaires 4, 5, 8, and 14 of nursing performance with low factor loading were removed and analyzed. As a result of factor analysis, three factors were extracted and labeled as application of nursing process, nursing competency, and nursing attitudes. Table 5 shows the results of factor analysis and reliability analysis in nursing work.

### 2.3. Data Collection and Processing Method

The data for this study were collected from nurses working at 2nd and 3rd general hospitals from 17 February to 18 April 2020 after gaining approval by the Institutional Bioethics Committee of CA University (1041078-201911-HR-351-01). The researchers explained to the participants that all responses to the questionnaire were anonymous and confidential; the answers were not intended to evaluate the individual, so there were no disadvantages to responding honestly to the questionnaire. Structural equation modeling was conducted to analyze the structural relationship among the factors set for nurses. SPSS 24.0 (IBM Corp., Armonk, NY, USA) and Amos 23.0 programs (IBM Corp., Armonk, NY, USA) were used to analyze the relationship between variables. In all analysis, the significance level was set at 5%. The analysis methods used in this study were frequency analysis, exploratory factor analysis, and correlation analysis. The validity of the measurement tool was verified by confirmatory factor analysis. The fitness index of the measurement model was used by the error mean square root (RMSEA), the incremental fit index (RMR), the fit index (GFI), the comparative fit index (CFI), and the non-standard fit index (TLI). To verify the fitness of the model, the statistical significance of the mediating effect of the parameters, set in this study, was verified through structural equation model analysis and the bootstrapping method.

## 3. Results

### 3.1. Descriptive Statistics and Correlations

The measurement items were verified for multivariate normality through descriptive statistical analysis. Table 6 shows the mean, standard deviation, skewness, and kurtosis for the factors communication ability, self-efficacy, self-leadership, and nursing performance.

Hu and Bentler [27] and Kim, Kim, and Hong [28] suggested checking whether the variables satisfy the multivariate normality distribution with skewness and kurtosis values. In other words, the absolute value of the skewness should be 3.0 or less and the absolute value of the kurtosis should be 8.0 or less.

Table 7 shows the results of analyzing the correlation between suggested communication ability, self-leadership, self-efficacy, and nursing performance variables. As shown in the results, the correlation between latent variables was statistically significant. The correlation coefficients presented were 0.453–0.753, which showed a good correlation overall.

### 3.2. Measurement Model Analysis

#### 3.2.1. Fit of Measurement Model

The confirmatory factor analysis was conducted to analyze the validity and reliability of the proposed measurement tool. Table 8 shows the fit indices of the measurement model in the measurement model analysis. The χ2 value has a characteristic that is sensitive to the size of the sample [28,29]. Other fit indices were also confirmed due to the characteristic of χ2. The results of the measurement model were χ2 = 94.846 (df = 51, *p* = 0.000). The results (CFI = 0.953, GFI = 0.924, TLI = 0.928, RMSEA = 0.072, RMR = 0.021) were found to meet the criteria for fitness presented in previous studies.

#### 3.2.2. Validity of the Measurement Model

The suitability indices CFI, GFI, TLI, RMSEA, and RMR of the set measurement model were found to satisfy the suitability criterion and additionally, the convergent validity of the measurement model was verified.

This analysis evaluated how well the observation variable explained the latent variable. As shown in Table 9, all factors except self-leadership were statistically significant, and satisfied the convergent validity. The standardization coefficient presented in Table 9 can provide the same meaning as factor loading in factor analysis. In this study, the standardization coefficient of each path presented was 0.50 or more, except for self-leadership and significance (C.R.), which was more than 1.965, meaning that the convergent validity was verified.

### 3.3. Structural Model Verification

#### 3.3.1. Verification of Model Fit

**Figure 1:**
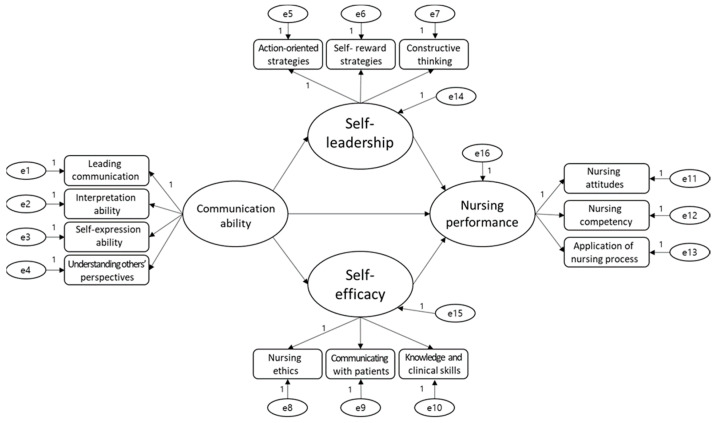
Structural Model.

The fitness of the model was verified; the structural model establishing the relationship between the communication ability, self-leadership, self-efficacy, and nursing performance was appropriate. Figure 1 shows the structural model according to the relationship among factors.

The structure model comprised four latent variables and 13 observed variables. Table 10 shows the fit of the established structural equation model. The analysis result of the measurement model shows that χ2 = 87.051 (df = 50, *p* = 0.000). In addition, the other fitness indexes, CFI = 0.960, GFI = 0.931, TLI = 0.938, RMSEA = 0.067, and RMR = 0.019, were found to meet the criteria for suitability presented in previous studies.

#### 3.3.2. Verification of Direct Effect

The fitness index of the established structural model was found to satisfy the criteria for conformity, and the direct effect was verified through the path coefficient and CR value between latent variables in the proposed study model. Table 11 shows the results.

First, as a result of verifying the relationship between communication ability and self-leadership, the standardized path coefficient of 1.049 and the CR value of 4.969 were found to have a statistically significant effect at the significance level of 0.001. Second, as a result of verifying the relationship between communication ability and self-efficacy, the standardized path coefficient 0.899 and CR value were found to have a statistically significant effect at the significance level of 0.001. Third, as a result of verifying the relationship between self-leadership and nursing performance, the standardized path coefficient −0.263 and CR value −0.357 were found to have no statistically significant effect. Fourth, as a result of verifying the relationship between self-efficacy and nursing performance, the standardized path coefficient 0.464 and CR value were 3.300, which was found to have a statistically significant effect at the significance level of 0.001. Fifth, as a result of verifying the relationship between communication ability and nursing performance, the standardized path coefficient −0.185 and CR value −0.267 were found to have no statistically significant effect.

### 3.4. Verification of the Mediation Effect

In the research model, self-leadership and self-efficacy were confirmed through direct, indirect, and total effects in the relationship between nurses’ communication ability and performance. Self-leadership had no indirect effect on the relationship between communication ability and nursing performance, and the mediating effect of self-efficacy had a confirmed statistical significance. The statistical significance was examined using the bootstrapping method provided by the Amos 23.0 program, and the results are shown in Table 12.

In the relationship between communication ability and nursing performance, the indirect effect of self-efficacy was 0.417 (0.899 0.464), which was statistically significant at the significance level 0.001. In other words, the communication ability indirectly affected nursing performance through the medium of self-efficacy perceived by the nurse. The communication ability of nurses working in the 2nd and 3rd general hospitals did not directly affect the nursing performance. This could have a complete mediating effect on nursing performance through self-efficacy.

## 4. Discussion

The purpose of this study was to empirically verify a model that would show a structural relationship between nurses’ communication ability, self-leadership, self-efficacy, and nursing performance. This information is essential because nurses are the core personnel of medical service institutions. The mediating effect of self-efficacy, which was a mediator variable, was also verified. The results of the study are discussed in detail below.

First, the communication ability of nurses had a positive effect on self-leadership and self-efficacy. In a study of nurses working in general hospitals, Song Gwai [2] explained that communication skills and self-leadership were closely related factors, and mentioned that communication skills grew higher as they age. Some also argued that communication skills are an important factor that directly or indirectly influences their leadership [18]. In addition, communication ability was explained as an important factor to increase the reliability of a nurse’s workplace and as a factor that can improve self-direction [2,16,17,30,31,32]. Son and Sung [6] revealed that communication ability is a variable that directly affects self-efficacy. In the study on the relationship between health beliefs and self-efficacy, Hong, Kim, and Suh [33] explained health beliefs as cognitive factors of individuals, and revealed that these health beliefs are helpful factors for participation in health behaviors and influence self-efficacy.

Second, self-efficacy had a positive effect on nursing performance. As a factor that can help improve nurse ability, several studies have argued for the importance of nurses’ self-efficacy [7,19,34]. Tracey [35] mentioned the difference between the general group and the control group according to the medical education experience of US nursing college students. In the case of the group who received this education, self-efficacy and satisfaction increased due to increased confidence. Through these results, the necessity of further education programs to improve the confidence and efficacy of nurses is identified. However, self-leadership and communication ability did not have a positive effect on nursing performance. In particular, first-time nurses with low clinical experience had many difficulties in establishing self-leadership, which supports the results of this study. However, in studies involving general companies and employees, self-leadership was recognized as a factor influencing nursing performance [36,37,38,39]. The difference between a nursing organization and general organization can be found in the organizational culture, that is, the nursing organizational culture is more rigid than the general organization. Barton [40] mentioned that communication and leadership are complementary in research on situations that can be missed and overlooked in the context of nursing leadership and communication.

Third, self-efficacy had a mediating effect in the relationship between communication ability and nursing performance. Son and Sung [6] revealed reveal that self-efficacy has a mediating effect on the relationship between communication ability and turnover intention, which is consistent with the results of this study. In addition, it can be seen that the communication ability of nurses does not directly lead to personal nursing ability [36,41,42]. In other words, as the self-efficacy increased through education, the individual’s nursing ability increased. Based on these findings, hospital officials should use a variety of education and support systems to improve their self-efficacy by increasing their confidence to improve hospital services and develop nurse competency.

## 5. Conclusions

The purpose of this study was to investigate the relationship among nurses’ communication ability, self-leadership, self-efficacy and nursing performance. The conclusions are as follows.

First, in the established model, physical fitness was verified with variables such as communication ability, self-efficacy, and nursing performance of nurses. As a result of model validation, the model’s fitness figures met the appropriate judgment criteria presented in previous studies, so the model between nurse communication skills, self-leadership, self-efficacy, and nursing outcomes was suitable for prediction. Second, the relationship between communication ability and self-leadership had a statistically significant effect, but the relationship between self-leadership and nursing performance had no statistically significant effect. In addition, it was found that self-efficacy had a statistically significant effect on the relationship between nursing outcomes. However, there was no statistically significant effect on the relationship between communication ability and nursing performance. Third, in the relationship between communication ability, an independent variable, and nursing performance, a dependent variable, it was confirmed that self-efficacy showed a significant result as a mediating variable.

The limitation of this study is that although the indicators for evaluating individual competences of nurses may differ, it is thought that the classification of nursing performance ability into performance ability, attitude, and nursing process may not be sufficient for accurate evaluation. Therefore, it is necessary to develop specific and objective tools in the future. In addition, since the response rate of nurses at the 2nd hospital participating in this research survey is much higher than that of the 3rd hospital, it may not be possible to generalize the results of this study. Based on the research results, in order to increase the understanding and competence of nurses, the core of efficient hospital service provision, it is necessary to change the education and nursing organization culture of hospitals in various ways.

## Figures and Tables

**Table 1:** Participant’s demographic characteristics.

Characteristics		Frequency (*n*)	Rate (%)
Gender	Male	11	6.5
	Female	157	93.5
Age	20~29	39	23.2
	30~39	50	29.8
	40~49	41	24.4
	50~	38	22.6
Married	Single	78	46.4
	Married	90	53.6
Academic records	College degree	90	53.6
	Bachelor’s degree	78	46.4
Types of medical institutions	Secondary hospital (General hospital)	142	84.5
	Third hospital (Advanced general hospital)	26	15.5
Clinical Experience	<3 years	33	19.6
	3~7 years	43	25.6
	7~11 years	39	23.2
	>11 years	53	31.5
Department	Internal medicine ward	96	57.1
	Surgical ward	31	18.5
	Pediatric ward	4	2.4
	Special department	37	22.0
Position	General nurse	145	86.3
	Charge nurse	17	10.1
	Head nurse	6	3.6
Annual Income (1,000,000 won /(1000 US dollar)	<30/(25)	46	27.4
	30~40 /(25~34)	65	38.7
	40~50 /(34~42)	45	26.8
	>50/(42)	12	7.1
Total		168	100

**Table 2:** Descriptions and factor loadings of communication ability and reliability.

Factor Name	Factor Variable	Factor Loading	% of Variance (Eigen Value)	Communality	Reliability (Cronbach’s Alpha)
Understanding others’ perspectives	comm16	0.827	29.096 (3.783)	0.688	0.779
	comm14	0.783		0.665	
	comm15	0.751		0.659	
	comm13	0.651		0.527	
Interpretation ability	comm 2	0.835	13.686 (1.779)	0.709	0.731
	comm1	0.770		0.685	
	comm 4	0.626		0.651	
Self-expression ability	comm 7	0.809	11.499 (1.495)	0.682	0.658
	comm6	0.683		0.614	
	comm 5	0.501		0.509	
Leading communication	comm10	0.740	8.875 (1.154)	0.727	0.639
	comm9	0.725		0.664	
	comm11	0.569		0.531	
Total		63.156			

Kaiser–Meyer–Olkin (KMO) measure of sampling adequacy = 0.700. Bartlett sphericity test = 646.215, df = 78, Sig. = 0.000.

**Table 3:** Descriptions and factor loadings of self-leadership and reliability.

Figure.	Factor Variable	Factor Loading	% of Variance (Eigen Value)	Communality	Reliability (Cronbach’s Alpha)
Constructive thinking	leadership12	0.796	33.568 (3.693)	0.669	0.807
	leadership13	0.776		0.608	
	leadership11	0.772		0.634	
	leadership10	0.760		0.678	
Self-rewarding strategies	leadership8	0.804	18.357 (2.019)	0.688	0.788
	leadership9	0.768		0.672	
	leadership7	0.751		0.683	
	leadership6	0.684		0.620	
Action-oriented strategies	leadership4	0.869	12.250 (1.348)	0.793	0.652
	leadership3	0.823		0.701	
	leadership2	0.538		0.613	
Total		64.175			

Kaiser-Meyer-Olkin (KMO) measure of sampling adequacy = 0.744. Bartlett sphericity test = 674.725, df = 55, Sig. = 0.000.

**Table 4:** Descriptions and factor loadings of self-efficacy and reliability.

Factor Name	Factor Variable	Factor Loading	% of Variance (Eigen Value)	Communality	Reliability (Cronbach’s Alpha)
Knowledge and clinical skills	efficacy4	0.865	50.226 (5.525)	0.840	0.898
	efficacy2	0.845		0.781	
	efficacy3	0.831		0.799	
	efficacy6	0.735		0.676	
Communicating with patients	efficacy10	0.826	12.667 (1.393)	0.736	0.838
	efficacy11	0.823		0.712	
	efficacy12	0.745		0.656	
	efficacy9	0.668		0.634	
Nursing ethics	efficacy14	0.767	8.772 (0.965)	0.744	0.754
	efficacy16	0.767		0.643	
	efficacy15	0.714		0.658	
Total		71.665			

Kaiser–Meyer–Olkin (KMO) measure of sampling adequacy = 0.744. Bartlett sphericity test = 674.725, df = 55, Sig. = 0.000.

**Table 5:** Descriptions and factor loadings of nursing performance and reliability.

Factor Name	Factor Variable	Factor Loading	% of Variance (Eigen Value)	Communality	Reliability (Cronbach’s Alpha)
Application of nursing process	performance 11	0.858	54.244 (5.424)	0.840	0.895
	performance 12	0.826		0.778	
	performance 10	0.826		0.785	
	performance 13	0.706		0.673	
Nursing competency	performance 2	0.865	11.332 (1.133)	0.822	0.855
	performance 1	0.814		0.755	
	performance 3	0.750		0.706	
Nursing attitudes	performance 9	0.407	9.364 (0.936)	0.509	0.751
	performance 7	0.880		0.852	
	performance 6	0.784		0.774	
Total		74.940			

Kaiser–Meyer–Olkin (KMO) measure of sampling adequacy = 0.861. Bartlett sphericity test = 989.783, df = 45, Sig. = 0.000.

**Table 6:** Descriptive statistics of observed variables (*n* = 168).

Measurement Variables	Mean	Standard Deviation	Skewness	Kurtosis
Leading communication	3.685	0.628	0.072	−0.120
Self-expression ability	4.040	0.542	0.076	−0.480
Interpretation ability	3.621	0.581	0.158	0.055
Understanding others’ perspectives	3.325	0.704	0.066	−0.472
Action-oriented strategies	3.582	0.644	−0.126	−0.283
Self-rewarding strategies	3.781	0.622	0.023	−0.245
Constructive thinking	2.911	0.682	−0.299	0.659
Nursing ethics	3.787	0.682	0.108	−0.838
Communicating with patients	3.933	0.562	0.202	−0.698
Knowledge and clinical skills	3.980	0.592	0.007	−0.634
Nursing attitudes	3.833	0.650	0.050	−0.669
Nursing competency	4.065	0.545	−0.171	0.292
Application of nursing process	3.988	0.601	−0.218	−0.789

**Table 7:** Correlations of latent variables.

Latent Variable	1	2	3	4
Communication ability	1			
Self-leadership	0.453 **	1		
Self-efficacy	0.526 **	0.480 **	1	
Nursing performance	0.498 **	0.462 **	0.753 **	1

** *p* < 0.01.

**Table 8:** Measurement model fit indices set.

Criteria	χ2	df	*p*	CFI	GFI	TLI	RMSEA	RMR
Model fit	94.846	51	0.000	0.953	0.924	0.928	0.072	0.021
Criteria for fitness				≥0.90	≥0.90	≥0.90	≤0.08	≤0.05

Note: CFI (comparative fit index), GFI (goodness of fit index), TLI (Turker-Lewis index), RMSEA (root mean square error of approximation), RMR (root mean square residual).

**Table 9:** The results of the confirmatory factor analysis.

Directions		Estimate (*p*)	Standardization Factor (β)	Standard Error (S.E.)	Critical Ratio (C.R.)
Leading communication	Communication ability	1.000	0.524		
Self-expression ability		0.723	0.460	0.169	4.270 ***
Interpretation ability		0.858	0.586	0.169	5.080 ***
Understanding others’ perspectives		0.888	0.523	0.187	4.753 ***
Action-oriented strategies	Self-leadership	1.000	0.013		
Self-rewarding strategies		0.565	0.581	0.216	0.149
Constructive thinking		0.766	0.714	0.926	0.148
Nursing ethics	Self-efficacy	1.000	0.720		
Communicating with patients		0.941	0.714	0.102	9.182 ***
Knowledge and clinical skills		1.274	0.797	0.124	10.297 ***
Nursing attitudes	Nursing performance	1.000	0.795		
Nursing competency		0.821	0.719	0.082	9.995 ***
Application of nursing process		1.090	0.804	0.095	11.524 ***

*** *p* < 0.001.

**Table 10:** The fit indices of measurement model.

Criteria	χ2	df	*p*	CFI	GFI	TLI	RMSEA	RMR
Model fit	87.051	50	0.000	0.960	0.931	0.938	0.067	0.019
Criteria for fitness				≥0.90	≥0.90	≥0.90	≤0.08	≤0.05

**Table 11:** Standardized direct effects of the model.

Directions	Estimate (*p*)	Standardization Factor (β)	Standard Error (S.E.)	Critical Ratio (C.R.)
Communication ability → Self-leadership	1.512	1.049	0.304	4.969 ***
Communication ability → Self-efficacy	0.899	0.899	0.257	4.935 ***
Self-leadership → Nursing performance	−0.263	−0.263	0.875	−0.357
Self-efficacy → Nursing performance	0.464	0.464	0.532	3.300 ***
Communication ability → Nursing performance	−0.185	−0.185	1.185	−0.267

*** *p* < 0.001.

**Table 12:** Mediated effect analysis.

Directions	Direct Effects	Indirect Effects	Gross Effects
Communication ability → Self-efficacy	0.899	-	0.899
Self-efficacy → Nursing performance	0.464	-	0.464
Communication ability → Nursing performance	-	0.417 ***	0.417

*** *p* < 0.001.
